# Sustainable Tannin Gels for the Efficient Removal of Metal Ions and Organic Dyes

**DOI:** 10.3390/gels9100822

**Published:** 2023-10-17

**Authors:** Ann-Kathrin Koopmann, Caroline Ramona Ehgartner, Daniel Euchler, Martha Claros, Nicola Huesing

**Affiliations:** 1 Department of Chemistry and Physics of Materials, Paris Lodron University of Salzburg, 5020 Salzburg, Austria; ann-kathrin.koopmann@plus.ac.at (A.-K.K.);; 2 Salzburg Center for Smart Materials, 5020 Salzburg, Austria; 3 Escuela de Ingeniería Química, Pontificia Universidad Católica de Valparaíso, Valparaíso 2362854, Chile

**Keywords:** adsorption, biosorbent, porous materials, tannin, metal complexation

## Abstract

The usage of a highly efficient, low-cost, and sustainable adsorbent material as an industrial wastewater treatment technique is required. Herein, the usage of the novel, fully sustainable tannin-5-(hydroxymethyl)furfural (TH) aerogels, generated via a water-based sol–gel process, as compatible biosorbent materials is presented. In particular, this study focusses on the surface modification of the tannin biosorbent with carboxyl or amino functional groups, which, hence, alters the accessible adsorption sites, resulting in increased adsorption capacity, as well as investigating the optimal pH conditions for the adsorption process. Precisely, highest adsorption capacities are acquired for the metal cations and cationic dye in an alkaline aqueous environment using a carboxyl-functionalized tannin biosorbent, whereas the anionic dye requires an acidic environment using an amino-functionalized tannin biosorbent. Under these determined optimal conditions, the maximum monolayer adsorption capacity of the tannin biosorbent ensues in the following order: Cu^2+^ > RB > Zn^2+^ > MO, with 500, 244, 192, 131 mg g^−1^, respectively, indicating comparable or even superior adsorption capacities compared to conventional activated carbons or silica adsorbents. Thus, these functionalized, fully sustainable, inexpensive tannin biosorbent materials, that feature high porosity and high specific surface areas, are ideal industrial candidates for the versatile adsorption process from contaminated (heavy) metal or dye solutions.

## 1. Introduction

**Figure 1:**
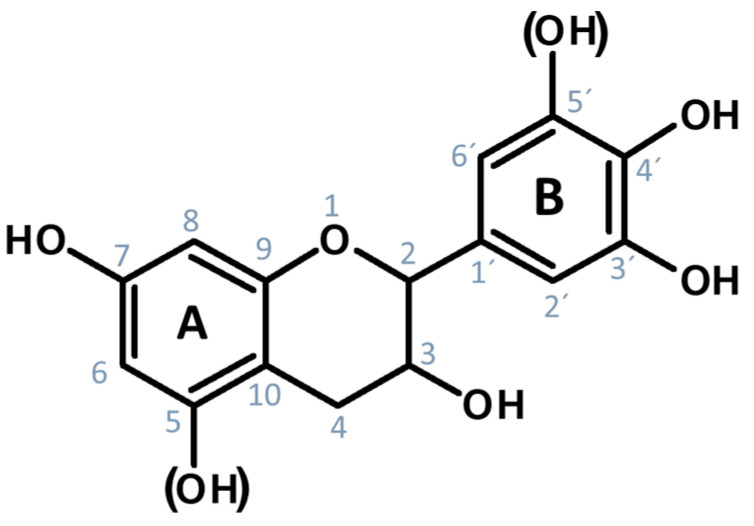
The monomeric flavonoid unit of condensed tannins, which is usually connected to adjacent units at positions 4 and 6 or 8.

Over the past decades, water pollution with heavy and critical metal ions as well as with organic (textile) dyes has become a worldwide concern. Hence, the treatment of contaminated wastewater is of high importance [1,2]. Versatile technologies based on physicochemical processes, such as membrane separation [3], chemical precipitation [4], electrode position [5], and adsorption [6,7,8], have been conventionally employed [9]. However, the mentioned techniques imply high costs and require a high heavy metal ion concentration, except for adsorption, which, in turn, represents a cost-effective technique with ease of operation as well as a high rate of recyclability [6,10]. Generally, adsorption is achieved by concentrating the adsorbate from a fluid phase onto the adsorbent’s surface or the adsorbent’s pores, and it can be classified into two types, namely, physi- and chemi sorption [1,11,12]. Conventional adsorbent materials include activated carbons [13], alumina [14], or silica [15], however, their generation often requires high processing temperatures [8,11,16,17]. In regard to building a circular economy by using eco-friendly materials, natural biopolymer adsorbent materials (referred to as biosorbents) have gained increasing interest in wastewater treatment due to their low costs, ease of processing, and environmental and economic sustainability, as well as their potential for regeneration [11,18,19,20]. Among others, tannins, which are low-cost, abundant, natural polyphenols, and are available as waste products from the paper/wood industry, show great potential as biosorbents [11,12,21]. In particular, their ease of conversion into insoluble matrices as well as their natural affinity to ionic dyes and metals in wastewaters benefits their performance as biosorbents [11,12,22]. The metal adsorption mechanism of condensed tannins, whose monomeric flavonoid repetition unit is shown in Figure 1, relies on the presence of the ortho-dihydroxyl group (3′-4′ site) of their B-ring, which chelates in an ion exchange reaction, (metal) ions to the B-rings adjacent hydroxyl groups [11,23,24,25]. The structure of the tannins B-ring depicts a vital role in the formation of the metal–tannate complexes, whereas a catechol-type B-ring (two adjacent hydroxyl groups) exhibits a weaker adsorption capacity for ions, compared to a pyrogallol-type B-ring (three adjacent hydroxyl groups) [24]. This is explained by the stabilization of the chelates in the presence of an additional third hydroxyl group [26]. Hence, condensed tannins with a pyrogallol-type B-ring, such as mimosa tannin [27], are supposed to have higher adsorption capacities.

There are several studies in literature dealing with the adsorption capacity of tannin-based materials to remove heavy metals, such as copper [18,22,28,29], zinc [18,22,30], lead [18,22,28,29], and chromium [29,31,32,33,34], as well as organic dyes, such as methylene blue (MB) [30] from aqueous solutions [11,25]. However, these research papers predominantly deal with single metal biosorption, whereas limited attention is given to the adsorption of multi-metal-ion systems, which albeit rather describe a freshwater sample [22]. The tannin-based adsorbent materials discussed in the literature, furthermore, have different forms, such as tannin foams, gels, resins, or are on three-dimensional structures as immobilized tannins. Speaking of tannin gels, they are produced in an insolubilization process, whereby the water-soluble tannin is polymerized in a reaction with an aldehyde, yielding a crosslinked tannin gel that can be used as a biosorbent [11,12]. Most commonly, toxic and carcinogenic formaldehyde is used as a crosslinking agent [22,28,31,35], however, in accordance with the biosorbents’ sustainability, its usage should be avoided [36]. Although tannins have already been studied as biosorbent materials, there are very limited studies concerning a tannin biosorbent material, that is synthesized using only renewable precursor in a water-based system.

Therefore, this study describes the usage of a fully sustainable class of tannin aerogels, synthesized using the mimosa tannin, which is a waste product in the paper industry, as well as the biobased crosslinker 5-(hydroxymethyl)furfural, as competitive, green adsorbent material. Hence, its applicability as a biosorbent, enabling the replacement of commercially available adsorbent materials, is evaluated. In particular, the adsorption capacities to different metal ions (copper and zinc) as well as textile dyes (rhodamine B and methylene orange) in regard to the tannin gels’ surface functionalization, adsorption kinetics over time, and initial pH value are addressed in detail.

## 2. Results and Discussion

### 2.1. Characterization of Monolithic Tannin Gels and Their Functionalization

Adsorption of heavy metals and organic dye molecules from wastewater by using biosorbents is already well discussed in the literature. The focus, typically, lies on the biosorbents’ surface functionalization benefitting the adsorption capacity [10,12]. However, there is still a lack of a low-cost, fully sustainable, and highly functional biosorbent for industrial applications. Hence, the generation of sustainable, monolithic aerogels, based on the tree-extract tannin and the biomass-derived crosslinker 5-HMF as well as their surface functionalization (carboxyl- and amino-modified) are hereinafter described and evaluated for their potential as adequate biosorbents. The synthesized tannin-5-HMF (TH) aerogels, pristine (TH-OH), carboxyl-functionalized (TH-COOH), and amino-functionalized (TH-NH_2_) are shown in Figure S1 and their globular-aggregated mesoporous network structures, visualized by SEM and TEM imaging, are depicted in Figure S2.

The diametric shrinkage after drying, the densities (bulk and skeletal), and, hence, the porosity of the TH aerogels varies significantly depending on the post-synthesis modification process (Table 1). By post-synthetically introducing functional groups and partially replacing the surface hydroxyl groups with either carboxyl or amino groups, the diametric shrinkage of the TH aerogels after supercritically drying was reduced. Whereas, after the introduction of amino groups, the lowest shrinkage, of 17%, compared to the highest shrinkage of the pristine TH gels (24%) was recorded. Concomitantly, the bulk density varied between the sample and shows lower values after the introduction of functional groups from 0.20 to 0.17 g cm^−3^. Furthermore, the porosity increases after surface functionalization, hence, the amino-modified TH gel features the highest porosity, of 88%. Overall, the post-modification process does not significantly influence the tannin network structure and the materials’ pore structure is further analyzed by nitrogen sorption analysis. 

The nitrogen sorption isotherms as well as the pore size distributions of the pristine and modified TH aerogels are presented in Figure S3. According to the IUPAC classification [37], the depicted isotherms are categorized as type IV, which implies a mesoporous structure. Precisely, this type of physisorption isotherm exhibits a characteristic hysteresis loop, provoked by capillary condensation processes within the mesopores (above *p/p_0_* of 0.5) [38]. The specific surface area (S_BET_), as depicted in Table 1, is slightly increased after surface functionalization from 441 m^2^ g^−1^ (pristine TH aerogels) to 509 m^2^ g^−1^ and 497 m^2^ g^−1^ for the carboxyl- and amino-functionalized TH aerogels, respectively. The hysteresis loop shows no saturation of N_2_ uptake at high *p/p_0_* values close to unity for the functionalized TH aerogels, suggesting the presence of macropores (>50 nm) [37]. Furthermore, the pore size distribution, evaluated by using the BJH method of the desorption branch, (Figure S3B) reveals an increase in the average pore size distribution after the post-modification, hence, the carboxyl- and amino-functionalized aerogels (12.7 and 11.8 nm, respectively) feature slightly larger pores than the pristine aerogels (11.3 nm). The changes in the pore size distribution, presumably, occur due to small structural changes within the network structure during the post-modification step.

**Figure 2:**
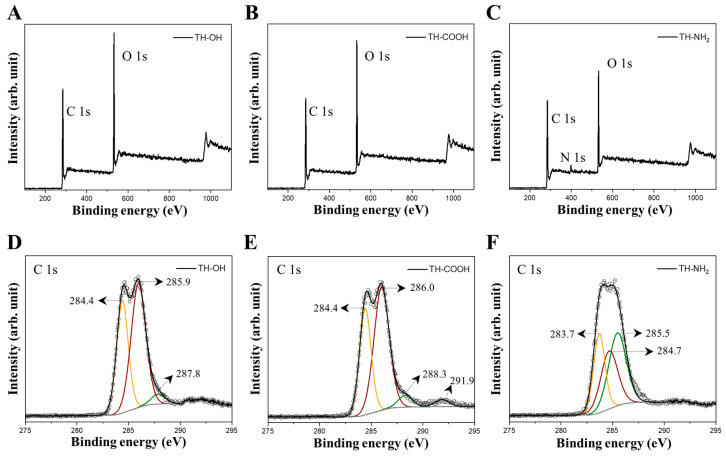
Overall survey XPS spectra (**A**–**C**) and high-resolution XPS spectra for C 1s (**D**–**F**) of the pristine, carboxyl-, and amino-functionalized TH aerogels, respectively.

The pristine tannin aerogel framework was grafted with EDTA (carboxyl groups) via a ring-opening transesterification process of the tannins’ hydroxyl groups by using EDTA anhydride. During the process, EDTA anhydride was hydrolyzed to EDTA [39,40]. Amination of condensed tannin using ammonia solution leads to a regioselective substitution at the pyrogallol B-ring, substituting hydroxyl groups with amino groups, yielding a 4′-amino-3′,5′-dihydroxybenzene B-ring [41,42]. In order to confirm the successful surface modification of the TH aerogels, FTIR measurements were performed (Figure S4). However, as the results were inconclusive, additional XPS analysis was conducted for a better understanding of the surface chemistry. Figure 2A–C depicts the overall survey XPS spectra of the pristine carboxyl- and amino-modified tannin aerogels, respectively; whereas Figure 2D–F illustrates the corresponding deconvoluted C 1s envelopes, whose peak assignments are listed in Table 2. The high-resolution XPS spectra of the deconvoluted O 1s and N 1s curves, as well as their peak assignments, are shown in Figure S5 and Table S1. The survey XPS spectrum of the pristine tannin gel (Figure 2A) indicates the presence of the elements carbon (C 1s, 285.1 eV) and oxygen (O 1s, 532.1 eV), which represent, together with hydrogen (non-detectable with the XPS technique), the chemical composition of tannins [43]. The deconvoluted C 1s spectrum of the pristine tannin aerogel (Figure 2D) reveals three main components at 284.4, 285.9, and 287.8 eV, which are associated with C=C, C–C/C–H/C–OH, and C=O/C–O, respectively [43,44,45,46,47]. The result matches the chemical structure of the pristine tannin, which features hydroxyl groups on its surface. Likewise, the carboxyl-modified tannin aerogel solely indicates the presence of carbon and oxygen atoms (Figure 2B). However, the C/O ratio slightly increased to 0.92, compared to the pristine tannin aerogel with a C/O ratio of 0.84, implying an increase in carbon content, which is, presumably, due to the introduction of carboxyl groups via EDTA esterification to the tannin aerogel. Precisely, the C 1s deconvoluted spectra of the carboxyl-modified tannin aerogels reveal in addition a supplementary peak at 288.3 eV, which are assigned to carboxylic acid and ester moieties (–COOH and O–C=O) [45]. Furthermore, a weak intensity π-π* shake-up satellite at 291.9 eV was detected, indicating the sp^2^ carbon aromatic constituent of the modified tannin sample [43]. It has to be noted that this shake-up satellite was also rudimentarily visual in the pristine tannin aerogel, however, it could not be deconvoluted due to its small contribution to the C 1s envelope. Overall, XPS analysis suggests successful partial surface replacement of hydroxyl groups with carboxyl groups after surface functionalization. Similarly, the survey XPS spectrum of the amino-modified tannin aerogel (Figure 2C) indicates the introduction of the amino groups, as, in addition to carbon and oxygen, also nitrogen is detected (N 1s, 399.0 eV). Furthermore, the C/O ratio is increased to 1.03, implying the replacement of some hydroxyl surface groups by amino groups. Nevertheless, hydroxyl groups still remain on the surface of the amino-modified tannin aerogels, as the C 1s deconvoluted envelope (Figure 2F) reveals, in addition to other peaks describing the tannin matrix, the peak located at 285.5 eV, describing alcohol moieties (C–OH). Furthermore, a minor peak can be surmised at approximately 287.7 eV, implying the presence of C-N moieties [45]. However, according to the high content of carbon and oxygen moieties in the tannin matrix, this contribution could not be deconvoluted. Nevertheless, the presence of nitrogen, precisely of amine moieties, is illustrated in the N 1s deconvoluted envelope of the amino-modified tannin aerogels (Figure S5D), which shows two components at 398.5 and 400.1 eV that can be assigned to C-N and N–H moieties, respectively [48].

Overall, the presented XPS data validated the successful surface functionalization of the pristine tannin aerogel as a partial replacement of the tannins’ surface hydroxyl groups with either carboxyl or amino groups.

### 2.2. Adsorption Studies

The accessibility of the tannin aerogels’ functional groups residing on the surface was investigated by adsorption experiments. Precisely, the influence of different surface functionalities (pristine, carboxyl-, and amino-functionalized) on the adsorption capacities for various adsorbates (zinc, copper, methyl orange (MO), and rhodamine B (RB)) is surveyed. Additionally, the influence of the pH value on the adsorption process, which, according to the literature, depicts an important factor [49,50], the adsorption kinetics, and potential recyclability was studied. Furthermore, the adsorption mechanism is explained in more detail in the supporting information.

#### 2.2.1. Effect of Initial pH 

**Figure 3:**
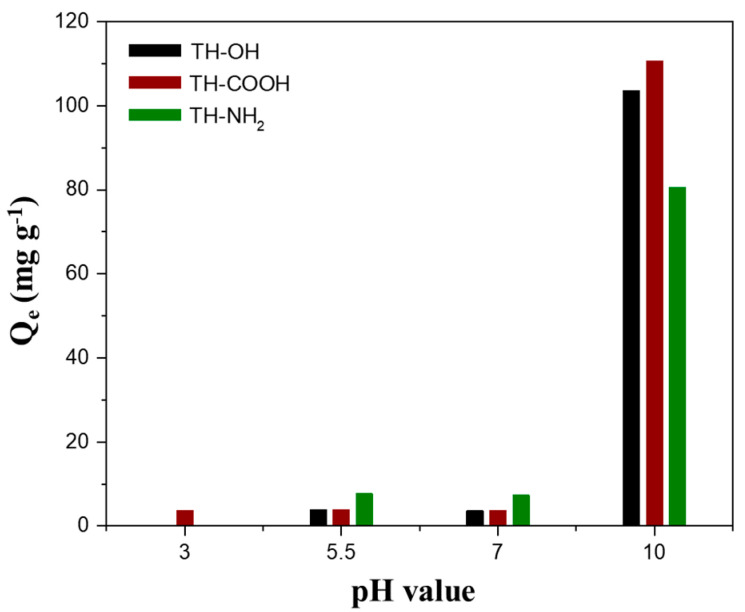
The pH-dependent adsorption capacities (Q_e_) of the pristine (black), carboxyl- (red), and amino-functionalized (green) tannin biosorbents for the adsorbate zinc with an initial zinc solution concentration of 200 mg L^−1^.

The pH value depicts a crucial factor, as it affects the interaction of adsorbent and adsorbate [24]. Figure 3 depicts the pH-dependent absorption behavior for zinc ions of the tannin monoliths, indicating a considerable increase in adsorbed zinc ions while processing in alkaline media (pH 10). Precisely, the p*K_a_* values of the tannins’ hydroxyl groups reside between 9.02 and 9.58. Thus, at basic conditions, the presence of the adsorbent’s active sites in their negatively charged dissociated forms is provoked [24]. Hence, the electrostatic attraction of positively charged zinc ions towards the tannins’ active sites is enhanced, benefiting the removal of the metal ions. In contrast, the p*K_a_* values of the carboxyl- and amino-moieties in the functionalized tannin biosorbent are significantly lower (p*K_a_* EDTA [51]: 0.26, 0.96, 2.60, and 2.76; p*K_a_* [52] 2-aminophenol: 4.78), implying deprotonation of these functional groups already at a lower pH value. However, the adsorbed mass of zinc ions on the functionalized monoliths is solely increased in an alkaline medium (pH > 7). A possible explanation for these observations depicts the formation of intermolecular hydrogen bonds in ortho-aminophenols [53] and 2,6-dihydroxybenzoic acid [54], preventing the dissociation of the amino- and carboxyl-group, respectively. Thus, after the deprotonation of the tannins’ hydroxyl groups (p*K_a_* of 9.02–9.58), the presence of intermolecular hydrogen bonds becomes obsolete, resulting in simultaneous deprotonation of the functional amino and carboxyl groups.

The removal of positively charged copper ions follows similar trends to the removal of zinc ions, with the same dependence on the pH value. However, in general, the affinity of tannin is higher for copper ions than for zinc ions, yielding higher adsorption capacities for copper ions already at neutral pH values. 

Furthermore, a pH-dependent behavior is also observed for the adsorption of organic textile dyes depending on the protonation/deprotonation of the adsorbent’s active sites, whereby cationic and anionic dyes behave differently. As expected, the negatively charged anionic dye methyl orange (MO) shows a stronger adsorption behavior in an acidic environment (pH 3) due to its attraction to the protonated adsorption sites of the tannin monolith. On the contrary, a positively charged cationic dye supposedly shows a better adsorption behavior in an alkaline medium, due to the attraction to the deprotonated adsorption sites, similar to the positively charged metal ions. This accounts for the investigated cationic dye rhodamine B (RB), where the adsorption capacity is increased when increasing the pH value from 7 to 11. However, just a minor increase is observed and the adsorbance performance at neutral pH only diminishes by 2–6%.

**Figure 4:**
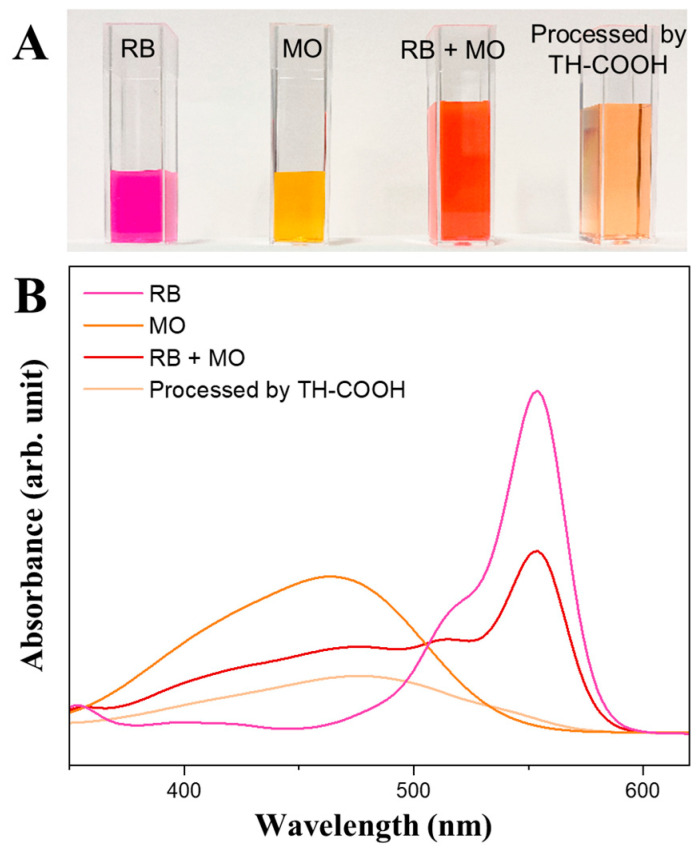
Images of the selective adsorption of RB from the RB/MO solution (pH 11) after two weeks (**A**) and the corresponding UV-vis spectra of the solutions before and after processing by TH-COOH (**B**).

Overall, depending on the charge of the metal ion or dye molecules, the deprotonation or protonation of the adsorbent’s active sites is required for a high adsorption capacity. Hence, due to the differences in pH-dependent adsorption behavior, the biosorbents can be used for selective adsorption of organic dyes (Figure 4). When mixing a cationic/anionic (RB/MO) dye solution at an initial pH value of 11, the tannin monolith (TH-COOH) shows excellent adsorption selectivity towards the positively charged RB molecules. According to UV-vis spectroscopy, the biosorbent exhibits a removal rate of 92% for the RB molecules (λ = 554 nm) and just a removal rate of 34% for MO molecules (λ = 464 nm) after two weeks of infiltration. 

#### 2.2.2. Effect of Surface Modification 

**Figure 5:**
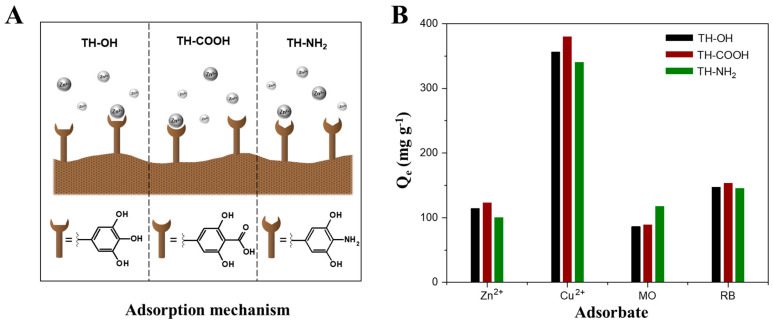
Schematic illustration of the surface functionalization dependent adsorption mechanism (**A**) and the maximum adsorption capacities (Q_e_) of the pristine (black), carboxyl- (red), and amino-functionalized (green) biosorbents for the adsorbates zinc ions (Zn^2+^, pH 10), copper ions (Cu^2+^, pH 10), methyl orange (MO, pH 3), and rhodamine B (RB, pH 11) at optimal pH conditions (**B**).

Additional to the pH dependency on the adsorption process, the influence of the type of adsorbents’ active sites, e.g., hydroxyl, carboxyl, or amino groups, was investigated (Figure 5A). The differences in adsorption capacity (Q_e_), determined at a specific initial concentration (300 mg L^−1^ for metal ions and 90 mg L^-1^ for organic dyes), for the pristine and functionalized tannin biosorbents on the four different adsorbates at optimal pH conditions (highest adsorbed mass of adsorbate), is represented in Figure 5B. For the metal ions Zn^2+^ and Cu^2+^ as well as the positively charged organic dye RB, the carboxyl-functionalized tannin yields the highest adsorption capacity. Whereas, the amino-functionalized tannin features the highest adsorption capacities for the negatively charged dye MO. This observation is explained by the different surface charges (zeta potentials) of the pristine and functionalized biosorbents. Precisely, a negative surface charge is enhanced by an increasing pH value, increasing polarity, and oxygen-containing functional groups [55]. Thus, amino-functionalized tannin features the least negative surface potential, implying the least strong electrostatic attraction towards positively charged metal ions and dye molecules and, hence, the least amount of adsorbed mass of these species [56]. Furthermore, differences in the amount of adsorbed adsorbates between the pristine (solely hydroxyl surface groups) and carboxyl-functionalized tannin, is explained by their differences in surface charges, relying on their different amounts of oxygen-containing functional groups. Precisely, during the post-modification process, tannins’ hydroxyl groups are partially modified in a transesterification process with EDTA anhydride. Hence, a functionalized hydroxyl group yields, presumably, three accessible carboxyl groups. Thus, the increase in oxygen-containing functional groups for the carboxyl-modified tannins results in a more negative surface charge and stronger attraction towards positively charged moieties. However, it has to be noted that the performance of pristine tannin is not significantly inferior to the high adsorption capacities of carboxyl-functionalized tannin. Thus, also pristine tannins, which are more cost-efficient and more sustainable, as they do not require a post-modification step, have to be considered for industrial applications.

Overall, the above-stated results allow for the evaluation of optimal process parameters regarding the initial pH and ideal surface modification, according to the adsorption capacities (Q_e)_ at a specific initial concentration. The positively charged adsorbates Zn^2+^ ions, Cu^2+^ ions, and RB, which perform better in an alkaline medium (pH 10–11), tend to yield better adsorption capacities for carboxyl-functionalized tannins. In contrast to that, the negatively charged adsorbates MO, beneficially processed in an acidic environment (pH 3), show greater capacities for amino-functionalized tannins. 

#### 2.2.3. Adsorption Kinetics 

The adsorption of the various adsorbates on the biosorbents was analyzed as a function of incubation time for initial adsorbate concentrations of 200 mg L^-1^ for metal ions and 50 mg L^-1^ for organic dyes. In order to characterize the adsorption kinetic data two reaction-based, first- and second-order kinetic models were applied.

Pseudo-first-order linear adsorption model [57]:

(1) $$\log\left(Q_{e}-Q_{t}\right)=\log\left(Q_{e}-K_{1}\right)$$

Pseudo-second-order linear adsorption model [57]:

(2) $$\frac{t}{Q_{t}}=\frac{1}{K_{2}Q_{e}^{2}}+\frac{t}{Q_{e}}$$

where Q_e_ (mg g^−1^) is the equilibrium adsorption capacity and Q_t_ (mg g^−1^) is the adsorption capacity at time t (h). K_1_ (1 h^−1^) and K_2_ (g mg^−1^ h^−1^) depict the pseudo-first- and -second-order rate constants, respectively.

**Figure 6:**
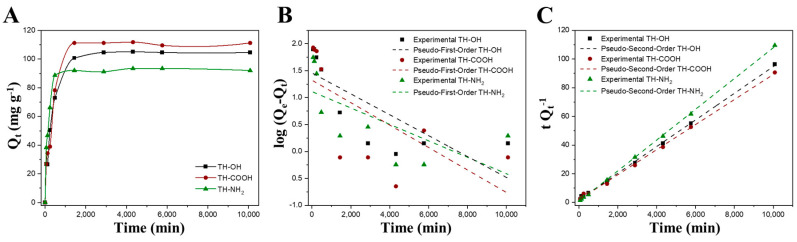
Adsorption kinetic curves for Zn^2+^ ions at pH 10 (**A**), pseudo-first-order linear kinetic model (**B**), and pseudo-second-order linear kinetic model (**C**) of the pristine (black), carboxyl- (red), and amino-functionalized (green) tannins.

Figure 6 representatively displays the adsorption kinetic of the monoliths for Zn^2+^ ions at pH 10, whereas the adsorption kinetic of Cu^2+^ ions, MO, and RB, are illustrated in the Supplementary Materials in Figures S6, S7, and S8, respectively. 

The adsorption reaction occurs rapidly in the beginning, as there is a high availability on the adsorbents’ active sites. Advancing the adsorption reaction, the active sites saturate with increasing incubation time until the adsorption equilibrium is reached [6]. Overall, the adsorption of metal ions takes place much faster with an adsorption equilibrium time t_eq_ of approximately 24 h, compared to an organic dye molecule, where t_eq_ for MO roughly accounts for 3 d and t_eq_ is not reached for RB after 7d. This is explained by the different sizes of metal ions vs. organic molecules. Precisely, the metal ions Zn^2+^ and Cu^2+^ (ionic radius of 0.074 and 0.073 nm, respectively) [58] feature a smaller relative size compared to the MO and RB dye molecules (molecular size of 1.19 nm × 0.67 nm × 0.38 nm and 1.44 nm × 1.09 nm × 0.64 nm, respectively) [59]. Hence, the metal ions are more easily conveyed to the adsorbents’ active sites due to less steric hindrance [7]. 

The pseudo-first- and pseudo-second-order kinetic parameters for the adsorption of Zn^2+^ ions, Cu^2+^ ions, MO, and RB on the biosorbents for the earlier determined optimal adsorption process conditions are depicted in Table 3 and all other kinetic parameters for other process conditions are given in Table S2. The adsorption of the metal ions Zn^2+^ and Cu^2+^ on the monoliths show the highest correlation coefficients (R^2^ > 0.999) for the pseudo-second-order-type kinetic adsorption reaction with good agreement of the experimental determined and calculated values for the adsorption capacities (Q_e_), indicating that chemisorption is the rate-determining step [24]. Thus, the adsorption rate is controlled by the availability of the adsorbents’ active sites rather than the adsorbates’ concentration [7]. In contrast to that, the adsorption of the anionic and cationic dyes rather follows the pseudo-first-order kinetic model (R^2^ > 0.982), suggesting that the adsorption reaction as a function of time directly correlates with the adsorbents’ saturation concentration (Q_e_) [60].

#### 2.2.4. Adsorption Isotherms at Optimal Process Conditions

In order to evaluate the gained results, the obtained experimental data were analyzed by the Langmuir [61] and Freundlich [62] models. Precisely, the Langmuir isotherm depicts the monolayer adsorption, assuming a homogeneous adsorbent surface structure with finite adsorption sites [24,57]:

(3) $$Q_{e}=\frac{Q_{m}\times {\;K}_{L}\times {\;C}_{e}}{1+K_{L}\times {\;C}_{e}}$$

whereas, the Freundlich isotherm assumes a heterogeneous surface, where a simultaneous mono- and multilayer adsorption can occur [24,57]:

(4) $$Q_{e}=K_{F}\times {\;C}_{e}^{1/n}$$

K_L_ is the Langmuir constant, related to the adsorption energy and K_F_ depicts the Freundlich constant, related to the adsorption capacity. Q_m_ (mg g^−1^) depicts the maximum monolayer adsorption capacity. 1/n portrays a predictor whether the adsorption is favorable.

**Figure 7:**
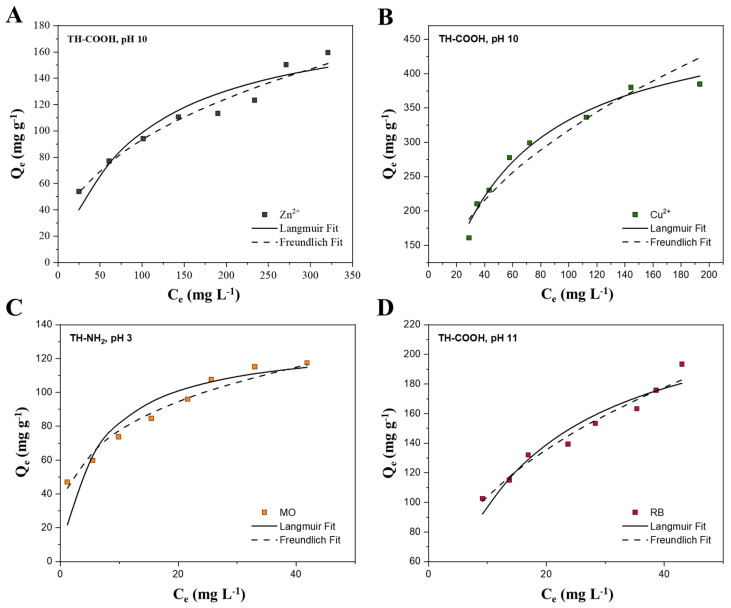
Adsorption isotherms at optimal processing conditions of Zn^2+^ ions (**A**), Cu^2+^ ions (**B**), MO (**C**), and RB (**D**).

The resultant adsorption isotherms, including a Langmuir and Freundlich fit for the determined optimal processing conditions for each investigated adsorbate, are illustrated in Figure 7 and their corresponding adsorption isotherm parameters are depicted in Table 4. All adsorption isotherms for the non-optimal processing parameters as well as their corresponding adsorption parameters are given in Figures S9–S12 and Table S3. For optimal processing conditions the maximum monolayer adsorption capacity Q_m_, determined by the Langmuir model, shows the sequence of Cu^2+^ >> RB > Zn^2+^ > MO with values ranging from 500 to 132 mg g^−1^, implying copper ions are, by far, most efficiently removed by the synthesized biosorbents.

According to the linear regression coefficients (R^2^), it is concluded that the adsorption isotherms for the different adsorbates rather follow the Freundlich model than the Langmuir model. This is attributed to the simultaneous formation of the adsorbate mono- and multilayer coverage on the adsorbent surface, implying heterogeneity of the material, where the adsorption sites have unequal adsorption energies [6]. The natural structural heterogeneity of condensed tannins, consisting of random combinations of different mono-flavonoid units, explains this inhomogeneous adsorption behavior [24]. However, the adsorption isotherm of copper ions on the carboxyl-functionalized tannin at pH 10 rather suggests a fit according to Langmuir. However, due to the high affinity of the tannin adsorbent within these processing conditions, it is assumed that a saturation of the adsorbent material is reached, hence, approaching the limits of the Freundlich fit. The 1/n factor from the Freundlich model between 0.1 and 1 describes an adsorption process under favorable conditions. The adsorption processes of Zn^2+^ ions, Cu^2+^ ions, MO, and RB are favorable.

The performance of the biosorbents towards Zn^2+^ ions, Cu^2+^ ions, MO, and RB is evaluated according to their maximum monolayer adsorption capacity Q_m_ in comparison to other adsorbent materials studied in the literature (Table 5). This study’s results exceed the adsorption capacities of conventional adsorbents, such as activated carbon and inorganic aerogels, and are comparable, or are even superior, to other biosorbents in most cases. The deviations in adsorption capacity may contribute to the experimental conditions, the materials’ specific surface area and the structural composition of the adsorbent as different functional surface groups feature different affinities towards metal ions and dye molecules [60]. However, overall, the synthesized monoliths depict convenient adsorption for different metal ions and organic dyes, and, together with their sustainable character, low costs, and ease of preparation, they are highly suitable for application on an industrial scale. 

#### 2.2.5. Regeneration Studies

**Figure 8:**
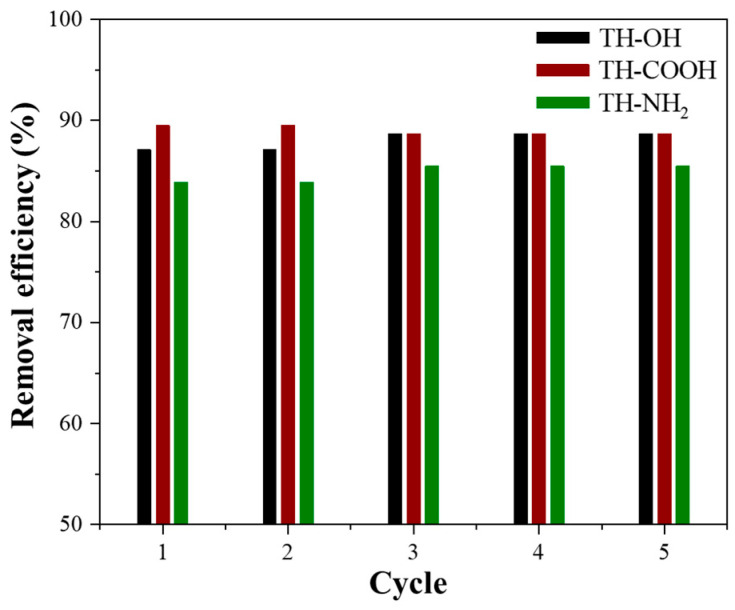
Performance of the pristine (black), carboxyl- (red), and amino-functionalized (green) tannins by five cycles of regeneration of Zn^2+^ ions.

The adsorbents’ regeneration and reuse are of high concern for practical application since a high adsorption capacity and good desorption performance has a favorable impact on the biosorbents overall costs, economic viability, and sustainability of the adsorption-based treatments [6,60]. Furthermore, the monolithic shape of the prepared tannin gels allows time-efficient recycling using an acidic desorption agent. Precisely, the monoliths feature promising recyclability over five cycles as illustrated in Figure 8 for the recovery of Zn^2+^ ions with exact recovery values given in Table S4. The removal efficiency of Zn^2+^ ions from an aqueous solution was constant over the first two cycles and then slightly increased and remained stable for the last three cycles. The increase in removal efficiency can be explained by the experimental setup, as, for starters, a dry monolith was used, which required more time to adjust to the liquid environment. After reaching the full adsorption capacity for zinc ions the removal efficiency remained steady at 89%, 89%, and 85% for the pristine, carboxyl-, and amino-functionalized monoliths, respectively.

The regeneration of the biosorbents for the adsorption of Cu^2+^ ions is, likewise, approximately stable over the period of five cycles, whereas it takes one cycle to reach the maximum adsorption capacity and a slight decrease in removal efficiency is observed after cycle three or four. Precisely, the removal efficiencies for Cu^2+^ ions ranged from 97–95%, 95–94%, and 94–93% for the pristine, carboxyl-, and amino-functionalized tannins, respectively (Figure S13 and Table S4). 

## 3. Conclusions

Low-cost, sustainable, non-toxic nanoporous aerogels were successfully synthesized in aqueous media, using the polyphenolic tree-extract mimosa tannin and biomass-derived crosslinker 5-(hydroxymethyl)furfural, and post synthetically surface functionalized. These nanoporous pristine and functionalized tannin-5-HMF aerogels feature low densities, high porosities, and high specific surface areas with marginal variations depending on their surface modification.

Within this study, the TH aerogels have been dried supercritically in order to precisely determine the adsorption capacity (mg per g of the biosorbent material). However, this drying step can be omitted by using solely the non-dried TH hydrogels for wastewater applications, and, hence, the overall production costs can be further reduced.

All in all, the adsorption process follows, according to the Freundlich model, a simultaneous mono- and multilayer adsorption, as the adsorptions sites have unequal adsorption energies due to the biosorbents’ heterogeneous structure. The metal adsorption process follows the pseudo-second-order kinetic model, suggesting a chemisorption process, whereas the organic dye adsorption process follows the pseudo-first-order kinetic model. The synthesized tannin monoliths show favorable adsorption of Zn^2+^ ions, Cu^2+^ ions, MO, and RB with optimal processing conditions for the metal ions and cationic dye in an alkaline medium and a carboxyl-functionalized tannin, whereas the anionic dye requires an acidic pH and an amino-functionalized tannin material. However, pristine tannins feature only slightly lower adsorption capacities towards Zn^2+^ and Cu^2+^ ions compared to the carboxyl-functionalized tannin aerogels. As inexpensive, sustainable biosorbents, generated without many processing steps being required, pristine tannins should also be acknowledged for their potential use on an industrial scale. The obtained results suggest that differences in the adsorbate retention occur due to different affinities of the metals or dyes for the adsorption sites of the tannins, whereas they show maximum adsorption capacities, according to Langmuir, at their optimal processing conditions in the following order: Cu^2+^ >> RB > Zn^2+^ > MO with 500, 244, 192, and 103 mg g^−1^, respectively. Furthermore, an effective regeneration capability for the metal ions over five cycles was shown.

Concluding, TH aerogels represent highly promising adsorbent materials for wastewater application due to their ease of generation, mild and aqueous reaction condition, sustainability (replacement of formaldehyde), and low costs. Their high adsorption capacities rely on the progressive complexation ability of tannins with metal ions and this study solely provides a new perspective of a green tannin material as an effective biosorbent. However, further research is required that focuses on the detailed analysis of the tannins’ adsorption mechanism, kinetics, and thermodynamics as well as their applicability in freshwater samples. 

## 4. Materials and Methods

### 4.1. Chemicals

The commercially available mimosa tannin “Weibull” extract was supplied by the Tanac company (Montenegro, Brazil). The 5-(hydroxymethyl)furfural (5-HMF, ≥ 95%) was acquired from AVA Biochem (Zug, Switzerland). Technical grade acetone (>99%), sodium hydroxide pellets (NaOH) and N,N-Dimethylformamide (DMF, 99.9%) were provided by VWR. Ammonium hydroxide (25% in H_2_O), zinc sulfate heptahydrate, Eriochrome^®^ Black T, ammonium chloride, Murexide, and methyl orange (MO) were obtained from Sigma Aldrich (St. Louis, MO, USA). Copper(II)sulfate (anhydrous) and acetic acid (glacial, 100%) were acquired from Merck (Darmstadt, Germany). Rhodamine B (RB) was provided by Alfa Aesar (Haverhill, MA, USA). Ethylenediaminetetraacetic acid disodium salt solution (0.1 M) was purchased from Fischer Scientific (Hampton, VA, USA). Ethylenediaminetetraacetic dianhydride (EDTAD, 98%) was supplied by ABCR (Karlsruhe, Germany).

### 4.2. Synthesis of Monolithic Tannin 5-HMF (TH) Aerogels

Tannin-5-HMF (TH) gels with a theoretical density of 0.1 g cm^−3^ were prepared by dissolving 2.5 g mimosa tannin in 25 ml deionized water (resistivity > 10 MΩ). The water-insoluble tannin fraction (approximately 4 wt%) was centrifuged off (4500 rpm for 15 min) and discarded. Then, 7.78 g 5-HMF was added to the remaining solution and the pH value was adjusted to pH 5 using 0.1M NaOH. After further stirring, the sol was filled inside glass vessels and placed in an oven at 80 °C to promote gelation. After seven days, the monolithic hydrogels were liberated from the glass vials. The gels were washed at least five times with acetone (roughly 50 mL) to ensure complete solvent exchange and extraction of the non-reacted species and byproducts. For determination of the metal and dye adsorption capacities, the wet gels were dried using supercritical extraction with CO_2_ (60 °C, 110 bar) [70]. For the applicability of the TH gels as biosorbents, however, drying is not required.

In order to surface functionalize the tannin gels’ hydroxyl groups with carboxyl or amino groups, the tannin gels were inserted into a 0.78 mmol EDTAD in DMF solution, catalyzed by glacial acetic acid, or into a 5M ammonia solution for 24 h, respectively.

### 4.3. Characterization of the Monolithic Gels

The monolithic TH gels were analyzed with duplicate measurements regarding their bulk densities ρ_b_, which were determined by weighing a sample of known dimensions and dividing this by the volume of the cylindrical sample according to the following formula:

(5) $$\rho_{b}=\frac{m}{r^{2}\pi h}$$

where m corresponds to the mass, r to the radius, and h to the height of the cylindrical gel. Moreover, to determine the skeletal density ρ_s_, helium pycnometry measurements were conducted on an ULTRAPYC 1200 e automatic density analyzer Quantachrome instrument. Hence, the overall porosity ϴ of the gels can be calculated according to the following formula:

(6) $$\theta \;=1-\left(\rho_{b}/\rho_{s}\right)$$

The pore textures of the monolithic gels, before and after surface functionalization, were investigated using nitrogen sorption analysis, performed on a Sy-Lab Micromeritics ASAP 2420 surface area and porosity analyzer (Norcross, GA, USA). After degassing the samples at 80 °C for 24 h under vacuum, the nitrogen sorption isotherms were recorded at −196 °C with duplicate measurements. The specific surface area S_BET_ is determined according to the Brunauer, Emmett, and Teller method and the pore size distribution for the nitrogen isotherm’s desorption branch has been analyzed by using the Barrett, Joyner, and Halenda method [71,72].

The XPS spectra were acquired using a Kratos Analytical Axis Supra instrument (Manchester, UK) with a passing energy of 20 eV for narrow and 80 eV for wide spectra. The emission current was 4.00 mA, with monochromatic Al Kα X-ray radiation and a hybrid lens mode was utilized. All the data were fitted calibrating the adventitious carbon at 284.7 eV, by CasaXPS software version 2.3.25 with Shirley background and Gaussian-Lorentzian (70–30%) line shape.

Fourier transform infrared (FTIR) spectroscopy was performed on the Vertex 70 instrument from Bruker (Billerica, MA, USA) with an attenuated total reflectance (ATR) module.

The morphology of the TH aerogels was analyzed by scanning electron microscopy (SEM), recorded with a Zeiss Ultra Plus instrument (Oberkochen, Germany), using an in-lens secondary electron detector as well as a varying acceleration voltage (5–10 kV). The samples were prepared on a carbon tape, followed by sputtering with gold. Transmission electron microscopy (TEM) images were taken with a JEOL JEM F200 microscope (Freising, Germany), which was equipped with a cold field emission source and which used a TVIPS F216 2k by 2k CMOS camera. Therefore, small pieces of the aerogel monoliths were ground and deposited on a copper grid. The TEM images were obtained using an electron acceleration voltage of 200 keV. 

UV-vis spectra were acquired using a PerkinElmer Lambda 750 device (Waltham, MA, USA).

### 4.4. Adsorption Experiments

#### 4.4.1. Equilibrium Adsorption Experiments

The adsorption performances of the tannin aerogels towards different heavy metal ions (Zn^2+^ and Cu^2+^) and organic dyes (RB and MO) were analyzed by equilibrium adsorption experiments at RT. 

Therefore, a given mass (roughly 0.02 g) of the pristine/modified tannin monolith was added to 50 ml of an aqueous heavy metal ion solution or organic dye solution of different concentrations (50 mg L^−1^–400 mg L^-1^). The pH of the solution was adjusted to either pH 7–11, using an ammonia buffer solution (ammonium chloride and ammonia 25%), or to pH 3 using a HCl/KCl buffer. The piece of the tannin monolith was left in solution for seven days and, afterwards, the metal ion or organic dye solution was separated from the solid adsorbent by decantation. Complexometric titration was used to determine the metal ion concentration and UV-vis spectroscopy was conducted to determine the dye concentration after separation, as described subsequently:

Zn^2+^-complexometric titration: The Zn^2+^ metal ion filtrate was titrated with a 0.01 M EDTA solution and the indicator Eriochrome^®^ Black T. The pH of the Zn^2+^ solution was adjusted to 10 by utilizing an ammonia buffer solution (ammonium chloride and 25% ammonia solution). 

Cu^2+^-complexometric titration: The Cu^2+^ metal ion filtrate was titrated with a 0.01 M EDTA solution and the indicator Murexide. The pH of the Cu^2+^ solution was adjusted to 10 by the use of an ammonia buffer solution (ammonium chloride and 25% ammonia solution). 

The adsorbed amount of heavy metal ions or dyes on the monolith can be calculated according to the subsequent equation [73]:

(7) $$Q_{e}=\frac{\left(C_{0}-C_{e}\right)\times \;V}{m}$$

where Q_e_ is the equilibrium adsorption capacity (mg g^−1^), C_0_ and C_e_ are the initial and the equilibrium concentrations of the heavy metal ion solutions/dye solutions (mg L^-1^), V is the volume of the heavy metal ion/dye solution (L), and m is the mass of the tannin aerogel used (g).

UV-vis spectroscopy: The concentration of the organic dye filtrate was investigated using UV-vis spectroscopy by acquiring the absorption spectra in the range of 200 to 800 nm. 

#### 4.4.2. Kinetic Adsorption Experiments

Roughly 0.02 g of the pristine/modified tannin monolith was added to 50 ml of an aqueous heavy metal ion solution (Cu^2+^ or Zn^2+^, 200 mg L^−1^) or organic dye solution (RB or MO, 50 mg L^−1^). The pH values of the solutions were adjusted to 10 (Cu^2+^ and Zn^2+^ solution) or 11 (RB solution) using an ammonia buffer solution (ammonium chloride and ammonia 25%), or to pH 3 (MO solution) using a HCl/KCl buffer. For kinetic adsorption experiments, the tannin monolith piece was kept in the mixture for certain time intervals, namely 1 h, 2 h, 4 h, 8 h, 24 h, 48 h, 3 d, 4 d, and 1 week. After decantation of the solid tannin adsorbent, the remaining metal ion concentration was determined by complexometric titration and the remaining dye concentration was determined by UV-vis spectroscopy, as described above.

#### 4.4.3. Selective Adsorption Experiments

A selective adsorption experiment was conducted, where 0.14 g of a tannin monolith were immersed in a mixture (pH 7) of 25 mL of rhodamine B solution (50 mg L^−1^) and 25 mL methyl orange solution (50 mg L^−1^). The monolith was left in the mixture to equilibrate over a period of seven days. After separation of the monolith from the mixture, the remaining dye concentration was determined via UV-vis spectroscopy.

#### 4.4.4. Reusability Experiments

In order to evaluate the reusability, a tannin monolith of roughly 0.28 g was added to 50 mL aqueous Zn^2+^ or Cu^2+^ heavy metal solution or dye solution, whose pH was adjusted to 10 using 2.5 mL of an ammonia buffer solution (pH 10) or a 5 mL of a 3M ammonium solution, respectively. The solutions were left to equilibrate over a time period of three days. Then, the tannin monolith was separated from the metal solution and was placed in 50 mL of a 1 M HCl solution for 24 h before washing four times with water. This process of metal adsorption, metal desorption, and washing was repeated five times. The metal ion concentration of the recyclability experiments was determined via complexometric titration as described above. 

## Figures and Tables

**Table 1:** Diametric shrinkage, bulk density, skeletal density, porosity, and specific surface area of the prepared tannin-5-HMF aerogels.

Sample	Shrinkage (%)	Bulk Density (g cm^−3^)	Skeletal Density (g cm^−3^)	Porosity ϴ (%)	S_BET_ (m^2^ g^−1^)	BJH Pore Diameter (nm)
TH-OH	23.5 ± 0.5	0.20 ± 0.01	1.48 ± 0.01	86.6 ± 0.6	441 ± 12	11.3 ± 1.6
TH-COOH	20.2 ± 1.2	0.19 ± 0.01	1.47 ± 0.02	87.3 ± 0.6	509 ± 6	12.7 ± 0.6
TH-NH_2_	17.1 ± 1.5	0.17 ± 0.02	1.41 ± 0.01	88.0 ± 1.0	497 ± 4	11.8 ± 0.1

**Table 2:** Peak assignments for the deconvoluted C 1s XPS envelopes of the pristine and modified tannin aerogels.

Sample	Element	Binding Energy (eV)	Assignment
TH-OH	C 1s	284.4	C=C
-	-	285.9	C–OH, C–C, C–H
-	-	287.8	C=O, C–O
-	-	-	-
TH-COOH	C 1s	284.4	C=C, C–C, C–H
-	-	286.0	C-OH
-	-	288.3	O–C=O, –COOH
-	-	291.9	π-π* satellite
-	-	-	-
TH-NH_2_	C 1s	283.7	C=C
-	-	284.7	C–C, C–H
-	-	285.5	C–OH

**Table 3:** Pseudo-first- and pseudo-second-order kinetic parameters for the adsorption of Zn^2+^ ions, Cu^2+^ ions, MO, and RB on the tannins at room temperature and the optimal pH value as well as functionalization.

	Linear Pseudo-First-Order			Linear Pseudo-Second-Order			
Adsorbate	K_1_ (min^−1^)	Q_e_ (mg g^−1^)	R^2^	K_2_ (g mg^−1^ min^−1^)	Q_e_ (mg g^−1^)	R^2^	Conditions
Zn^2+^	−2.0 × 10^−4^	21.1	0.675	4.0 × 10^−5^	114.9	0.999	TH-COOH, pH 10
Cu^2+^	−3.0 × 10^−4^	155.1	0.968	1.7 × 10^−5^	322.6	0.999	TH-COOH, pH 10
MO	−2.0 × 10^−4^	69.1	0.997	1.7 × 10^−5^	94.3	0.995	TH-NH_2_, pH 3
RB	−2.0 × 10^−4^	99.6	0.982	1.1 × 10^−6^	147.1	0.885	TH-COOH, pH 11

**Table 4:** Langmuir and Freundlich adsorption isotherm parameters for the adsorption of Zn^2+^ ions, Cu^2+^ ions, MO, and RB on the tannins at room temperature and the optimal pH value as well as functionalization.

	Langmuir			Freundlich			
Adsorbate	K_L_ (L mg^−1^)	Q_m_ (mg g^−1^)	R^2^	K_F_ (mg^1−n^ L^n^ g^−1^)	1/n	R^2^	Conditions
Zn^2+^	0.011	192.3	0.957	459.7	0.408	0.982	TH-COOH, pH 10
Cu^2+^	0.019	500.0	0.990	6308.1	0.427	0.965	TH-COOH, pH 10
MO	0.164	131.6	0.943	5206.0	0.279	0.985	TH-NH_2_, pH 3
RB	0.066	243.9	0.966	5735.9	0.385	0.983	TH-COOH, pH 11

**Table 5:** Comparison of maximum monolayer adsorption capacity Q_m_ of Zn^2+^ ions, Cu^2+^ ions, MO, and RB with other adsorbents, studied in the literature.

Adsorbent	Zn^2+^ Q_m_ (mg g^−1^)	Cu^2+^ Q_m_ (mg g^−1^)	MO Q_m_ (mg g^−1^)	RB Q_m_ (mg g^−1^)	Conditions	SSA (m² g^−1^)	Ref.
TH aerogel	192.3 *	500.0 *	131.6 ^+^	243.9 *	* TH-COOH, pH 10 ^+^ TH-NH_2_, pH 3	441–509	This work
Pine tannin gel	65.0	-	-	-	pH 7	2–6	[30]
Valonia tannin resin	35.5	45.4	-	-	pH 5	11–12	[22]
Phenolic foams based on tannins	29.1	46.5	-	-	pH 5–7	-	[36]
Chitosan/tannin/ montmorillonite film	-	-	57.4	-	pH 7	-	[63]
Chitosan–tannin paper	-	684.9	-	-	pH 9	-	[6]
Tannic acid functionalized graphene	-	-	-	201.0	pH 11	-	[64]
Activated carbon	3.9	6.8	94.6	61.0	pH 5.5	79–1170	[65,66,67,68]
Carbon aerogel	1.8	561.7	-	/	pH 6	544	[69]
Carboxyl-functionalized silica aerogel	111.0	78.0	-	154.0	pH 8	145	[7]

* TH-COOH, pH 10. ^+^ TH-NH_2_, pH 3.

## Data Availability

No new data were created or analyzed in this study. Data sharing is not applicable to this article.

## Supplementary Materials

The following supporting information can be downloaded at: https://www.mdpi.com/article/10.3390/gels9100822/s1, Figure S1: Photographs of the pristine (A), carboxyl-functionalized (B), and amino-functionalized (C) TH aerogels, illustrated on a 1 cm^2^ grid; Figure S2: Scanning electron micrographs (A–C) and corresponding transmission electron micrographs (D–F) of the pristine, carboxyl-, and amino-functionalized TH aerogels, respectively; Figure S3: Nitrogen adsorption (full symbols) and desorption (empty symbols) isotherms (A) and the pore size distribution (B) of pristine (black), carboxyl-functionalized (red), and amine-functionalized (green) TH aerogels; Figure S4: FTIR-ATR spectra of the pristine (black), carboxyl- (red), and amino-functionalized (green) TH aerogels; Figure S5: High-resolution XPS spectra for O 1s of the pristine, carboxyl-, and amino-functionalized TH aerogels (A-C, respectively) and high-resolution XPS spectrum for N 1s of the amino-functionalized gels (D); Table S1: Peak assignments for the deconvoluted O 1s and N 1s XPS envelopes of the pristine and modified TH aerogels; Figure S6: Adsorption kinetic curves for Cu^2+^ ions at a pH value of 10 (A), Pseudo-first-order linear kinetic model (B) and Pseudo-second-order linear kinetic model (C) of the pristine (black), carboxyl- (red), and amino-functionalized (green) tannins; Figure S7: Adsorption kinetic curves for MO dye molecules at a pH value of 3 (A), Pseudo-first-order linear kinetic model (B) and Pseudo-second-order linear kinetic model (C) of the pristine (black), carboxyl- (red), and amino-functionalized (green) tannins; Figure S8: Adsorption kinetic curves for RB dye molecules at a pH value of 11 (A), Pseudo-first-order linear kinetic model (B) and Pseudo-second-order linear kinetic model (C) of the pristine (black), carboxyl- (red), and amino-functionalized (green) tannins; Table S2: Pseudo-first and pseudo-second-order kinetic parameters for the adsorption of Zn^2+^ ions, Cu^2+^ ions, MO, and RB on the tannins at room temperature and the optimal pH value as well as non-optimal functionalization; Figure S9: Adsorption isotherms at pH 7 of Zn^2+^ ions on the pristine (A), carboxyl- (B), and amino-functionalized tannins(C) and adsorption isotherms at pH 10 of Zn2+ ions on the pristine (D) and amino-functionalized tannins (E); Figure S10: Adsorption isotherms at pH 7 of Cu^2+^ ions on the pristine (A), carboxyl- (B), and amino-functionalized tannins (C) and adsorption isotherms at pH 10 of Cu2+ ions on the pristine (D) and amino-functionalized tannins (E); Figure S11: Adsorption isotherms at pH 7 of MO on the pristine (A), carboxyl- (B), and amino-functionalized tannins (C) and adsorption isotherms at pH 3 of MO dye on the pristine (D) and carboxyl-functionalized tannins (E); Figure S12: Adsorption isotherms at pH 7 of RB dye on the pristine (A), carboxyl- (B), and amino-functionalized tannins (C) and adsorption isotherms at pH 11 of RB dye on the pristine (D) and amino-functionalized tannins (E); Table S3: Langmuir and Freundlich adsorption isotherm parameters for the adsorption of Zn^2+^ ions, Cu^2+^ ions, MO, and RB on the tannins at room temperature and the non-optimal processing condition; Table S4: Removal efficiency of the biosorbent for Zn^2+^ and Cu^2+^ ions after five cycles of regeneration; Figure S13: Performance of the pristine (black), carboxyl- (red), and amino-functionalized (green) tannins by five cycles of regeneration of Cu^2+^ ions.
