# Supplementary material for: Sustainable Tannin Gels for the Efficient Removal of Metal Ions and Organic Dyes
Source: Gels. 2023 Oct 17;9(10):822. doi: 10.3390/gels9100822 (PMC10606356; doi:10.3390/gels9100822)
Supplement: Supplementary file 1 [file gels-09-00822-s001.zip › gels-2628103-supplementary.pdf]

Supporting Information

Sustainable Tannin Gels for the Efficient Removal of Metal Ions and Organic Dyes

Ann-Kathrin Koopmann [1,2], Caroline Ramona Ehgartner [1,2], Daniel Euchler [1], Martha Claros [3] and Nicola Huesing [1,2,*]

[1] Department of Chemistry and Physics of Materials, Paris Lodron University of Salzburg, 5020 Salzburg, Austria; ann-kathrin.koopmann@plus.ac.at (A.-K.K.); caroline.ehgartner@gmail.com (C.R.E.); daniel.euchler@hotmail.com (D.E.)

[2] Salzburg Center for Smart Materials, 5020 Salzburg, Austria

[3] Escuela de Ingeniería Química, Pontificia Universidad Católica de Valparaíso, Valparaíso 2362854, Chile; marthaclarosvargas@gmail.com

* Correspondence: nicola.huesing@plus.ac.at

Number of Pages: 12

Number of Figures: 13

Number of Tables: 4

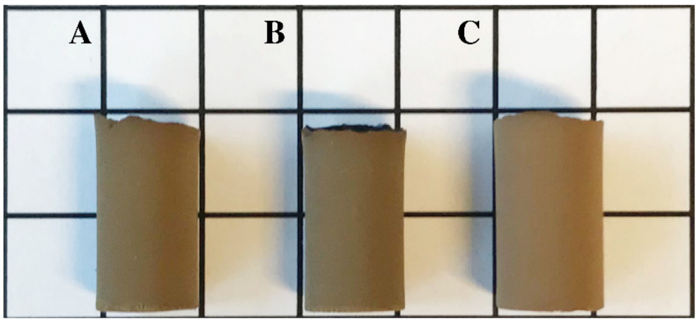

Figure S1 Photographs of the pristine (A), carboxyl-functionalized (B), and amino-functionalized (C) TH aerogels, illustrated on a 1 cm^2 grid.

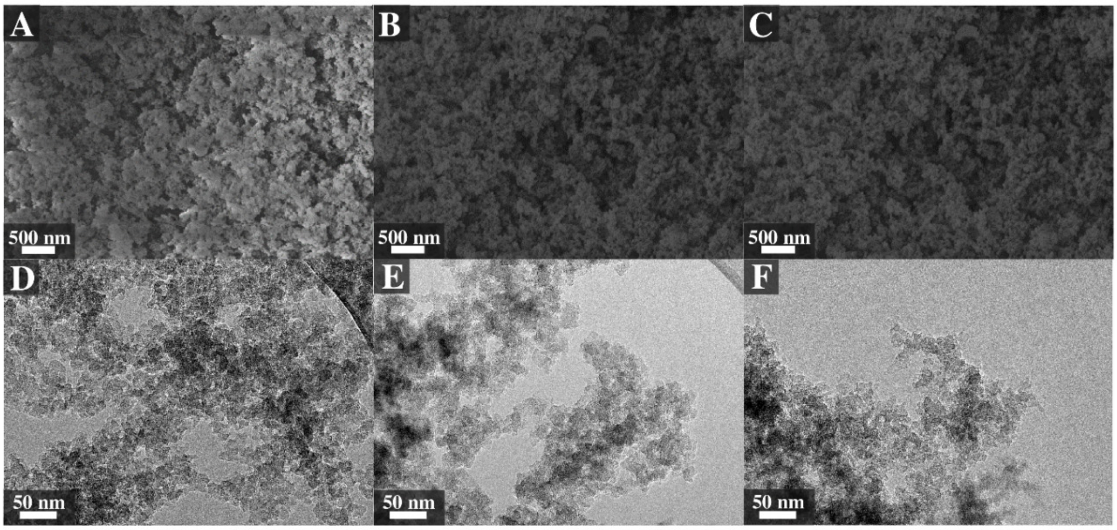

Figure S2 Scanning electron micrographs (A-C) and corresponding transmission electron micrographs (D-F) of the pristine, carboxyl-, and amino-functionalized TH aerogels, respectively.

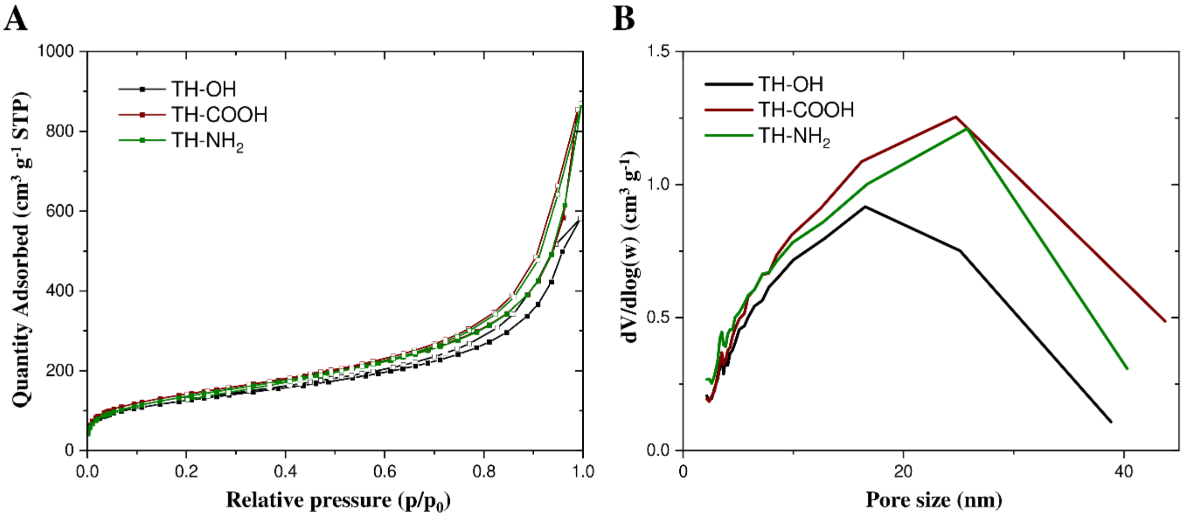

Figure S3 Nitrogen adsorption (full symbols) and desorption (empty symbols) isotherms (A) and the pore size distribution (B) of pristine (black), carboxyl-functionalized (red), and amine-functionalized (green) TH aerogels.

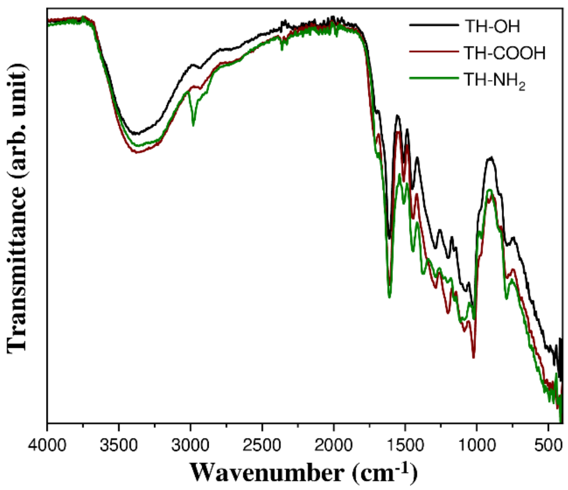

Figure S4 FTIR-ATR spectra of the pristine (black), carboxyl- (red), and amino-functionalized (green) TH aerogels.

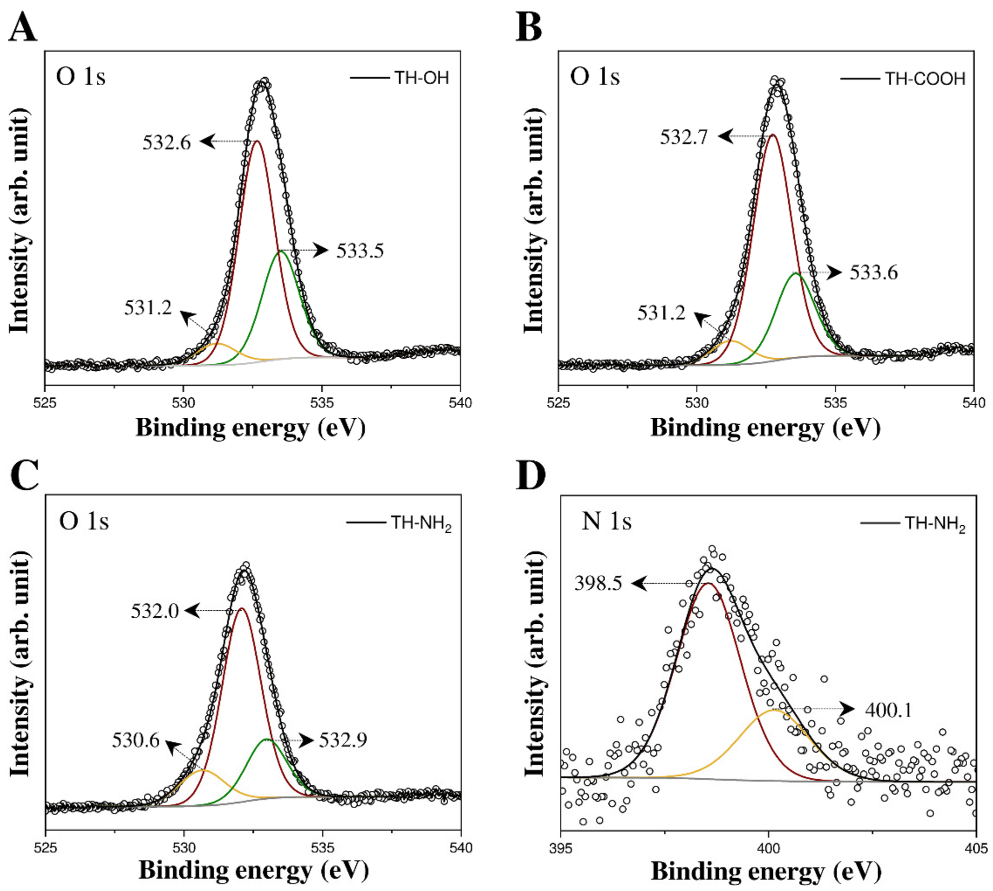

Figure S5 High-resolution XPS spectra for O 1s of the pristine, carboxyl-, and amino-functionalized TH aerogels (A-C, respectively) and high-resolution XPS spectrum for N 1s of the amino-functionalized gels (D).

Table S1 Peak assignments for the deconvoluted O 1s and N 1s XPS envelopes of the pristine and modified TH aerogels.

**Sample**	**Element**	**Binding energy (eV)**	**Assignment**
TH-OH	O 1s	531.2	C-O-H
		532.6	O-H
		533.5	O-C (aromatic)
TH-COOH	O 1s	531.2	O=C
		532.7	O-C
		533.6	O-C (aromatic)
TH-NH_2	O 1s	530.6	C-O-H
		532.0	O-H
		532.9	O-C
	N 1s	398.5	C-N
		400.1	N-H

Removal mechanism:

According to Oo and coworkers [1], the process of heavy metal ion removal from water includes various mechanisms, such as complexation, ion exchange as well as surface adsorption. Tannins have a great ability to remove metal ions from waste water due to their adjacent ortho-dihydroxyl groups in their B-rings, which can be either of catechol (two adjacent hydroxyl groups) or pyrogallol type (three adjacent hydroxyl groups). The structure of tannins is shown in the introduction section (Figure 1). The main adsorption mechanism relies on an ion exchange reaction, where metal cations replace the adjacent hydroxyl groups in order to from chelates. Furthermore, the oxygen on the phenolic group acts a strong Lewis base, forming coordination complexes with the metal ions (chemical entities, that are low in electrons). Divalent metal cations replace two protons from the tannins' B-ring adjacent hydroxyl groups, which in turn donate two electron pairs to the metal ion in order to form the metal-tannate chelates. The arrangement of the hydroxyl groups on the tannins B-ring plays a crucial role in the creation of these metal-tannate complexes. When the B-ring has a catechol-type, it has a lower ability to remove metal ions compared tannins with a pyrogallol-type B-ring. This difference is attributed to the enhanced stability of chelates in the presence of an additional third hydroxyl-group.[1] Please also refer to the review article from Koopmann and coworkers for detailed schematic representation of the tannin-metal complexation mechanism [2].

In this study, investigates the adsorption capacities of sustainable, porous tannin materials for the divalent metal cations Cu^{2+} and Zn^{2+} as well as the cationic dye rhodamine B (RB) and the anionic dye methyl orange (MO). The experiments thereby focus on the alteration of accessible adsorption sites by partial surface functionalization of the tannin biosorbent with carboxyl and amino groups. Additional focus remains on other influence factors such as pH of the solution as well as the investigation of the adsorption kinetics and reusability of the material. However, the investigation regarding the adsorption mechanism in more detail, by e.g. DFT calculations, is part of future work. This study solely focuses on the applicability of the sustainable tannin material as biosorbent and the effect of the surface functionalization on the adsorption capacities.

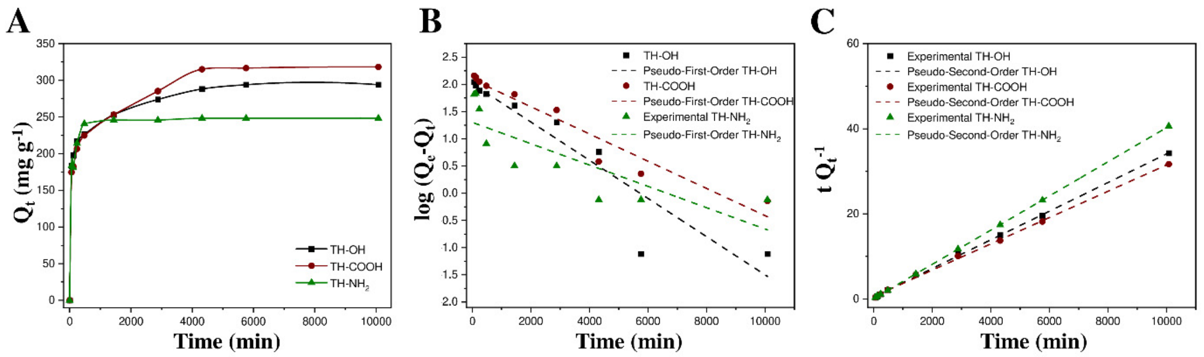

Figure S6 Adsorption kinetic curves for Cu^{2+} ions at a pH value of 10 (A), Pseudo-first-order linear kinetic model (B) and Pseudo-second-order linear kinetic model (C) of the pristine (black), carboxyl- (red), and amino-functionalized (green) tannins.

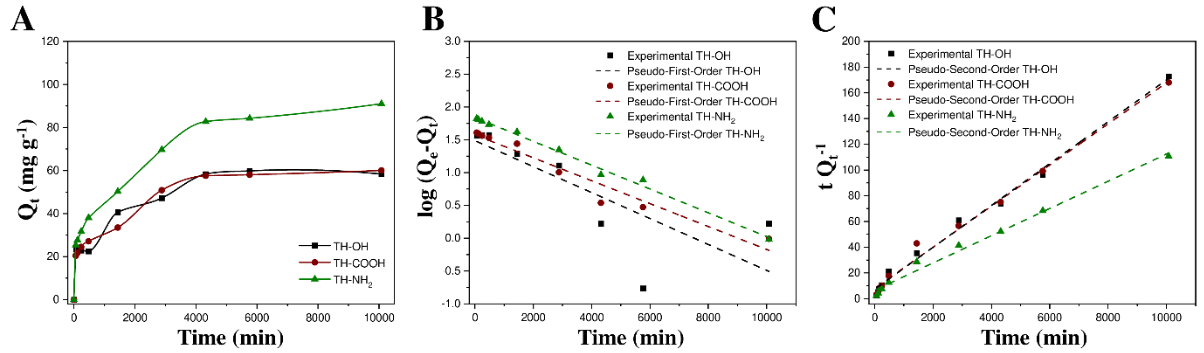

Figure S7 Adsorption kinetic curves for MO dye molecules at a pH value of 3 (A), Pseudo-first-order linear kinetic model (B) and Pseudo-second-order linear kinetic model (C) of the pristine (black), carboxyl- (red), and amino-functionalized (green) tannins.

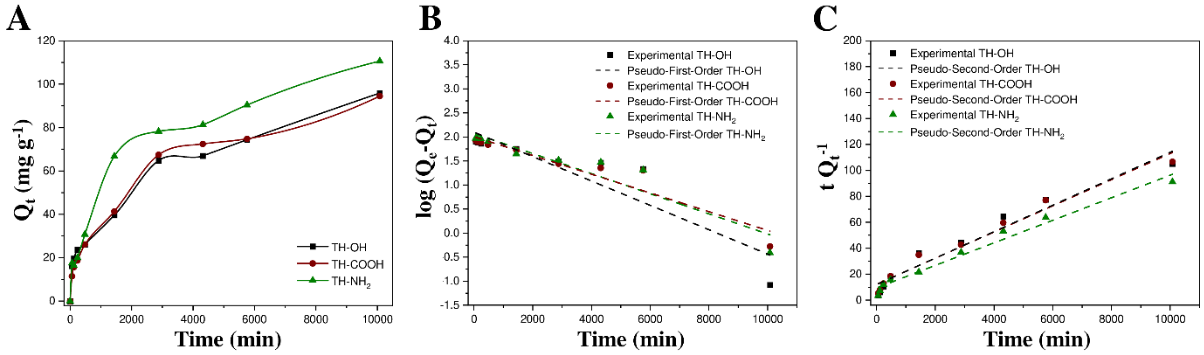

Figure S8 Adsorption kinetic curves for RB dye molecules at a pH value of 11 (A), Pseudo-first-order linear kinetic model (B) and Pseudo-second-order linear kinetic model (C) of the pristine (black), carboxyl- (red), and amino-functionalized (green) tannins.

Table S2 Pseudo-first and pseudo-second-order kinetic parameters for the adsorption of Zn^{2+} ions, Cu^{2+} ions, MO, and RB on the tannins at room temperature and the optimal pH value as well as non-optimal functionalization.

	Linear Pseudo First Order			Linear Pseudo Second Order			
**Adsorbate**	**K_1 [min^{-1}]**	**Q_e [$mg\ g^{-1}$]**	**R^2**	**K_2 [$g\ mg^{-1}\ min^{-1}$]**	**Q_e [$mg\ g^{-1}$]**	**R^2**	**Conditions**
**Zn^{2+}**	-2.0E-04	35.2	0.781	4.6E-05	107.5	1.000	TH-OH, pH 10
**Zn^{2+}**	-2.0E-04	13.0	0.672	1.8E-04	93.5	1.000	TH-NH_2, pH 10
**Cu^{2+}**	-5.0E-04	142.7	0.938	2.7E-05	294.1	1.000	TH-OH, pH 10
**Cu^{2+}**	-1.0E-03	59.0	0.814	1.4E-05	250.0	1.000	TH-NH_2, pH 10
**MO**	-2.0E-04	30.6	0.800	3.6E-05	61.3	0.996	TH-OH, pH 3
**MO**	-2.0E-04	37.5	0.975	3.2E-05	62.5	0.995	TH-COOH, pH 3
**RB**	-2.0E-04	75.9	0.976	1.8E-06	129.9	0.900	TH-OH, pH 11
**RB**	-2.0E-04	55.7	0.905	4.9E-06	95.2	0.980	TH-NH_2, pH 11

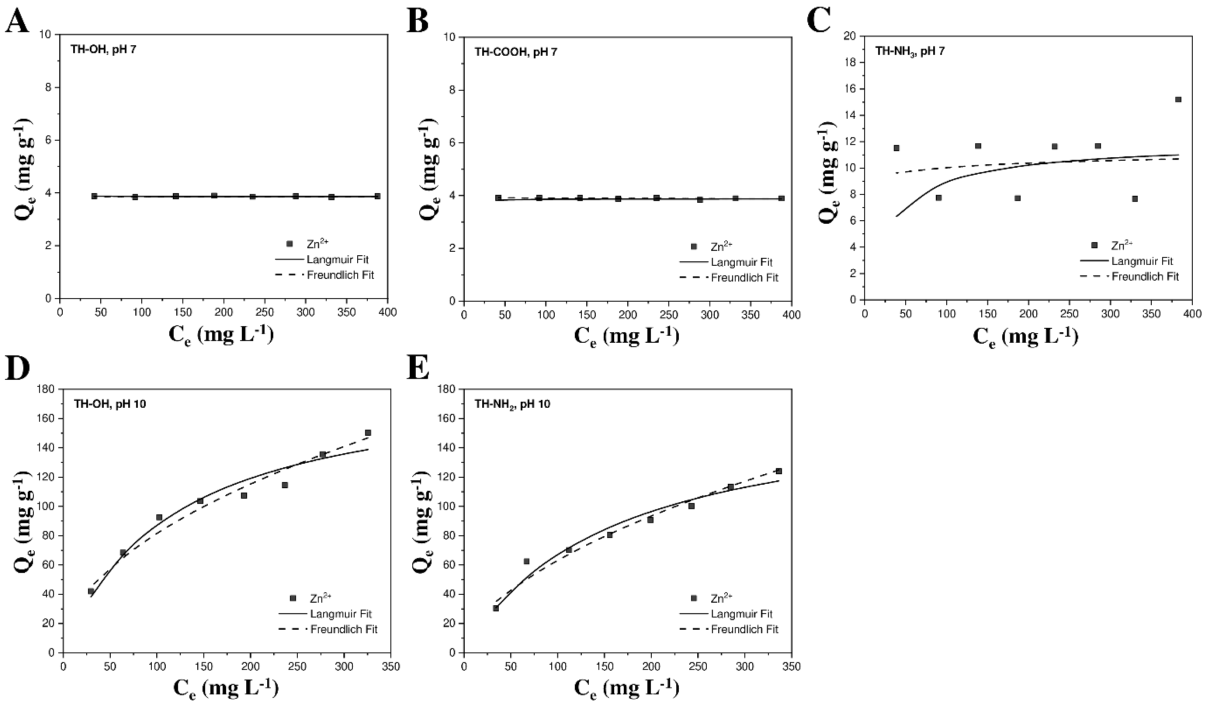

Figure S9 Adsorption isotherms at pH 7 of Zn^{2+} ions on the pristine (A), carboxyl- (B), and amino-functionalized tannins(C) and adsorption isotherms at pH 10 of Zn^{2+} ions on the pristine (D) and amino-functionalized tannins (E).

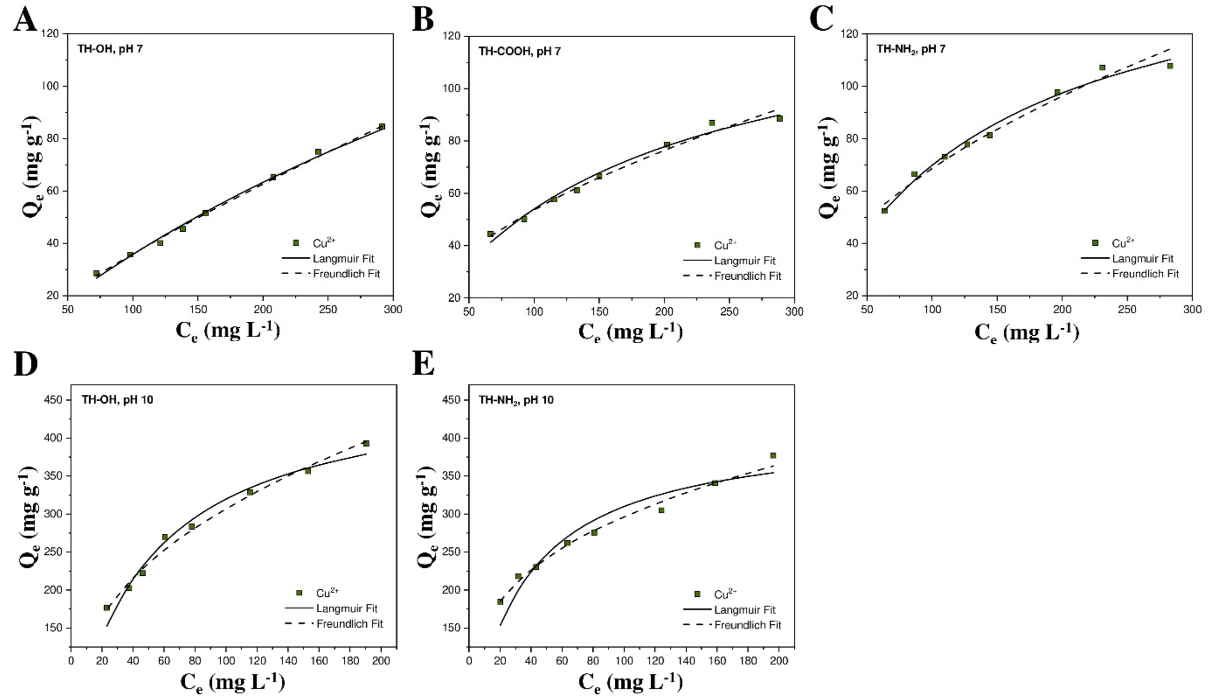

Figure S10 Adsorption isotherms at pH 7 of Cu^{2+} ions on the pristine (A), carboxyl- (B), and amino-functionalized tannins (C) and adsorption isotherms at pH 10 of Cu^{2+} ions on the pristine (D) and amino-functionalized tannins (E).

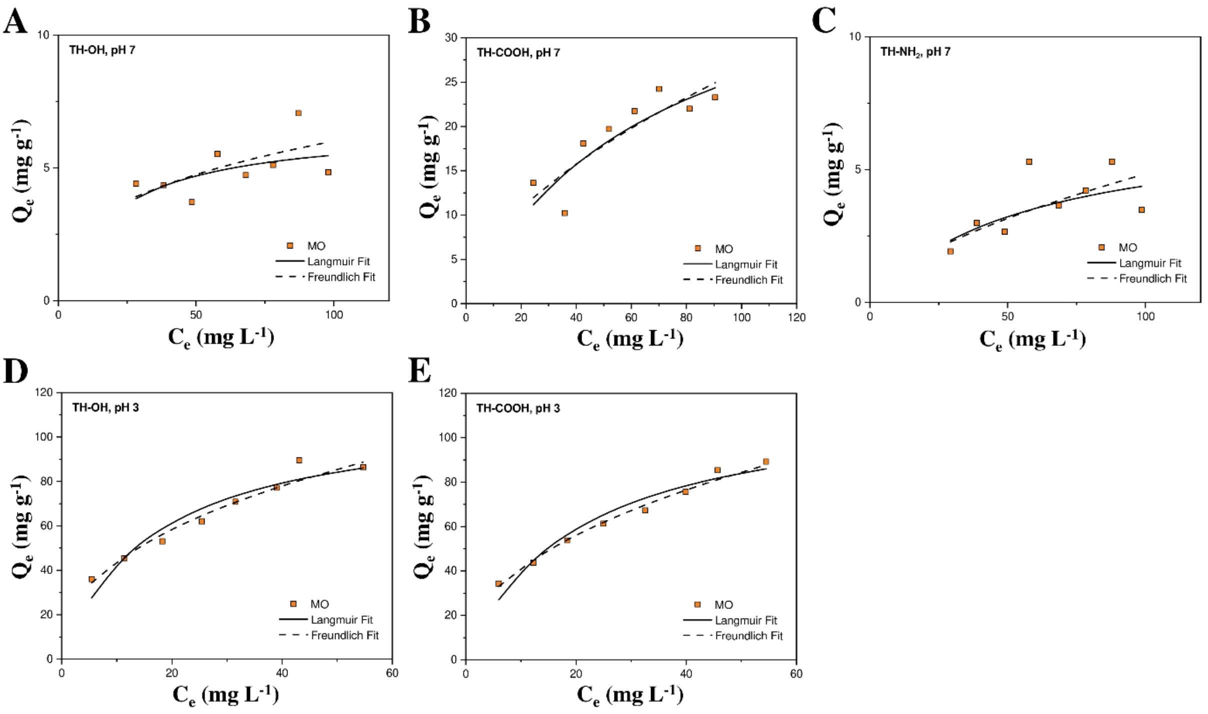

Figure S11 Adsorption isotherms at pH 7 of MO on the pristine (A), carboxyl- (B), and amino-functionalized tannins (C) and adsorption isotherms at pH 3 of MO dye on the pristine (D) and carboxyl-functionalized tannins (E).

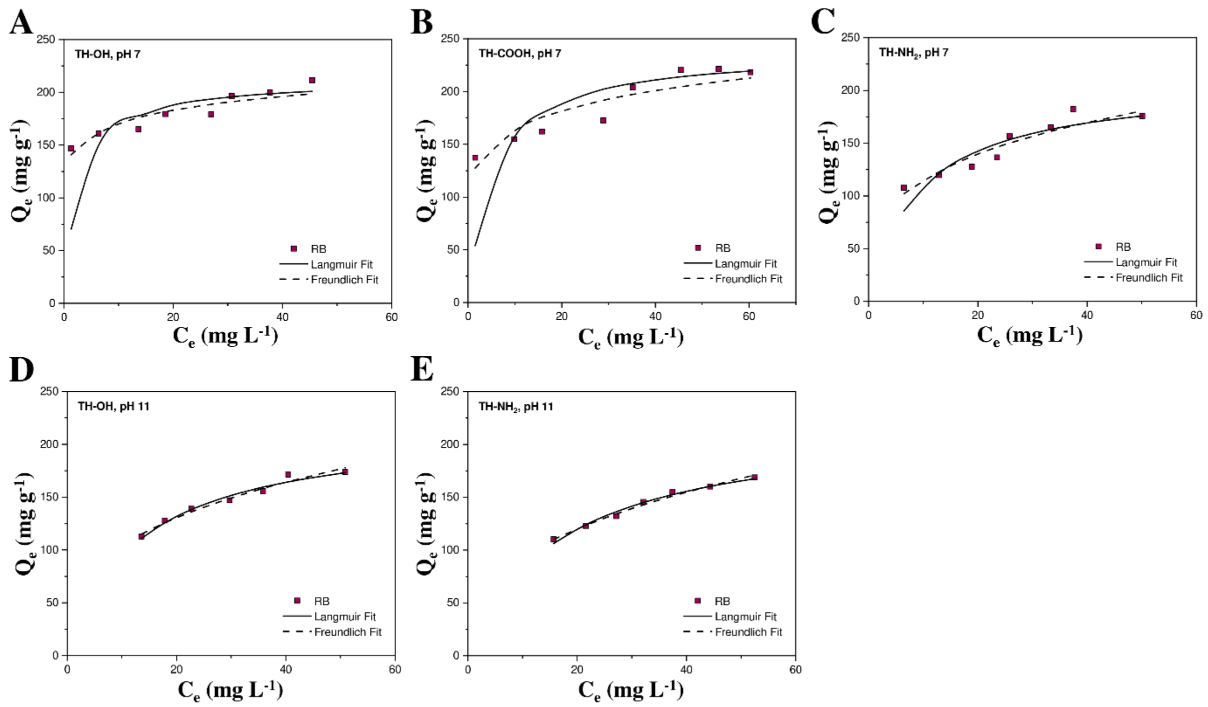

Figure S12 Adsorption isotherms at pH 7 of RB dye on the pristine (A), carboxyl- (B), and amino-functionalized tannins (C) and adsorption isotherms at pH 11 of RB dye on the pristine (D) and amino-functionalized tannins (E).

Table S3 Langmuir and Freundlich adsorption isotherm parameters for the adsorption of Zn^{2+} ions, Cu^{2+} ions, MO, and RB on the tannins at room temperature and the non-optimal processing condition.

	**Langmuir**			**Freundlich**			
**Adsorbate**	**K_L [L mg^{-1}]**	**Q_m [mg g^{-1}]**	**R^2**	**K_F [mg^{1-n} L^n g^{-1}]**	**1/n**	**R^2**	**Conditions**
**Zn^{2+}**	0.009	188.7	0.976	136.7	0.493	0.983	TH-OH, pH 10
**Zn^{2+}**	0.006	172.4	0.981	38.8	0.557	0.987	TH-NH_2, pH 10
**Zn^{2+}**	-7.038	3.9	0.064	22.5	0.000	0.036	TH-OH, pH 5.5
**Zn^{2+}**	1.836	3.9	-0.433	24.1	-0.005	0.485	TH-COOH, pH 5.5
**Zn^{2+}**	0.029	12.0	0.102	125.2	0.046	0.191	TH-NH_2, pH 5.5
**Cu^{2+}**	0.020	476.2	0.988	8855.2	0.387	0.995	TH-OH, pH 10
**Cu^{2+}**	0.029	416.7	0.966	21657.1	0.295	0.993	TH-NH_2, pH 10
**Cu^{2+}**	0.001	277.8	0.997	0.786	0.801	0.998	TH-OH, pH 5.5
**Cu^{2+}**	0.007	138.9	0.991	43.4	0.510	0.991	TH-COOH, pH 5.5
**Cu^{2+}**	0.008	161.3	0.992	95.1	0.489	0.988	TH-NH_2, pH 5.5
**MO**	0.060	112.4	0.960	688.0	0.412	0.979	TH-OH, pH 3
**MO**	0.050	117.6	0.976	504.1	0.443	0.994	TH-COOH, pH 3
**MO**	0.050	6.5	0.544	1.7	0.338	0.565	TH-OH, pH 7
**MO**	0.014	43.3	0.871	4.9	0.561	0.862	TH-COOH, pH 7
**MO**	0.018	6.9	0.672	0.1	0.611	0.807	TH-NH_2, pH 7
**RB**	0.077	217.4	0.986	7716.1	0.330	0.986	TH-OH, pH 11
**RB**	0.058	222.2	0.993	4965.9	0.365	0.994	TH-NH_2, pH 11
**RB**	0.373	212.8	0.818	83118.9	0.097	0.924	TH-OH, pH 7
**RB**	0.194	238.1	0.827	61858.6	0.139	0.919	TH-COOH, pH 7
**RB**	0.108	208.3	0.911	12746.8	0.279	0.945	TH-NH_2, pH 7

Table S4 Removal efficiency of the biosorbent for Zn^{2+} and Cu^{2+} ions after five cycles of regeneration.

Sample	Cycle	Removal Efficiency [%] Zn^{2+}	Removal Efficiency [%] Cu^{2+}
TH-OH	1	87.09	91.74
	2	87.09	96.81
	3	88.71	96.81
	4	88.71	95.22
	5	88.71	95.22
TH-COOH	1	89.51	88.43
	2	89.51	95.22
	3	88.71	95.22
	4	88.71	93.62
	5	88.71	93.62
TH-NH_2	1	83.87	93.73
	2	83.87	93.51
	3	85.48	93.39
	4	85.48	93.39
	5	85.48	93.39

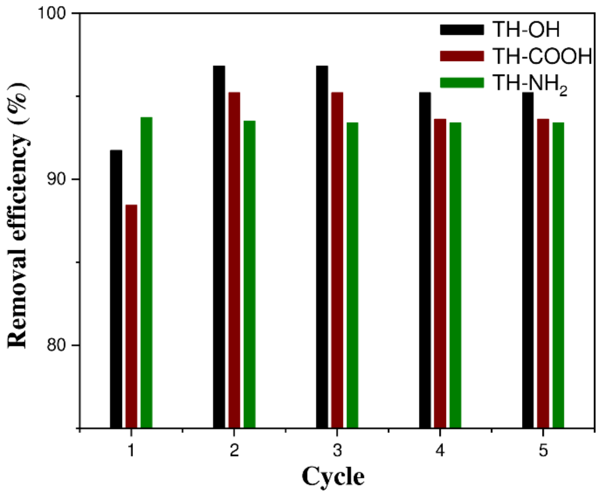

Figure S13 Performance of the pristine (black), carboxyl- (red), and amino-functionalized (green) tannins by five cycles of regeneration of Cu^{2+} ions.

References

1 Oo, C.W.; Kassim, M.J.; Pizzi, A. Characterization and performance of Rhizophora apiculata mangrove polyflavonoid tannins in the adsorption of copper (II) and lead (II). *Ind. Crops Prod.* **2009**, *30*, 152–161, doi:10.1016/j.indcrop.2009.03.002.

2 Koopmann, A.-K.; Schuster, C.; Torres-Rodríguez, J.; Kain, S.; Pertl-Obermayer, H.; Petutschnigg, A.; Hüsing, N. Tannin-Based Hybrid Materials and Their. *Molecules* **2020**, *25*, 4910, doi:10.3390/molecules25214910.
